# Can Environmental Stressors Determine the Condition of Ecological Plant Groups?

**DOI:** 10.3390/plants13111550

**Published:** 2024-06-04

**Authors:** Beata Koim-Puchowska, Piotr Kamiński, Piotr Puchowski, Anna Ossowska, Monika Wieloch, Mateusz Labudda, Halina Tkaczenko, Tadeusz Barczak, Alina Woźniak, Natalia Kurhaluk

**Affiliations:** 1 Department of Biotechnology, Kazimierz Wielki University, Ks. J. Poniatowski St. 12, PL 85-671 Bydgoszcz, Poland; koimpuchowska@ukw.edu.pl; 2 Division of Ecology and Environmental Protection, Department of Medical Biology and Biochemistry, Collegium Medicum in Bydgoszcz, Nicolaus Copernicus University in Toruń, M. Skłodowska-Curie St. 9, PL 85-094 Bydgoszcz, Poland; ania.ossowska@cm.umk.pl (A.O.); monika.wieloch@cm.umk.pl (M.W.); 3 Department of Biotechnology, Institute of Biological Sciences, Faculty of Biological Sciences, University of Zielona Góra, Prof. Z. Szafran St. 1, PL 65-516 Zielona Góra, Poland; 4 Government Forestry in Toruń, Zamrzenica Forestry District, Zamrzenica 1A, PL 89-510 Bysław, Poland; piotr.puchowski@torun.lasy.gov.pl; 5 Department of Biochemistry and Microbiology, Institute of Biology, Warsaw University of Life Sciences-SGGW, Nowoursynowska St. 159, PL 02-776 Warsaw, Poland; mateusz_labudda@sggw.edu.pl; 6 Institute of Biology, Pomeranian University in Słupsk, Arciszewski St. 22 B, PL 76-200 Słupsk, Poland; halina.tkaczenko@upsl.edu.pl (H.T.); natalia.kurhaluk@upsl.edu.pl (N.K.); 7 Department of Biology and Animal Environment, Faculty of Animal Breeding and Biology, Bydgoszcz University of Science and Technology, Hetmańska St. 33, PL 85-039 Bydgoszcz, Poland; tadbar@pbs.edu.pl; 8 Department of Medical Biology and Biochemistry, Collegium Medicum in Bydgoszcz, Nicolaus Copernicus University in Toruń, Karłowicz St. 24, PL 85-092 Bydgoszcz, Poland; al1103@cm.umk.pl

**Keywords:** disturbed environments, salinity, soil organic matter, halophytes, glycophytes, lipoperoxidation, proline, condition

## Abstract

There is still a need to investigate the relationships between glycophytes and halophytes and the many biotic and abiotic factors in their natural environments. Therefore, we study the effects of the type of environment on the ecophysiological responses and condition of the glycophyte Elder *Sambucus nigra* L., the macrophyte Common Reed *Phragmites australis* (Cav.) Trin. ex Steud., the facultative halophyte Weeping Alkaligrass *Puccinellia distans* (Jacq.) Parl, and the obligate halophyte Common Glasswort *Salicornia europaea* L. in a saline-disturbed anthropogenic region of central Poland. We analyzed the effects of salinity, acidity, and soil organic matter on shoot length, lipoperoxidation, and proline in roots and green parts, and evaluated plant responses to environmental disturbance, which allowed for the comparison of adaptation strategies. The studies were carried out in (1) “sodium production” (near sodium factories), (2) “anthropogenic environments” (waste dumps, agroecosystems, calcium deposits, post-production tanks), (3) “wetland environments” (near river channels and riparian areas), and (4) “control” (natural, unpolluted environments). Green parts of plants are better suited to indicate environmental stress than roots. Their higher structural MDA membrane damage is related to the transport of toxic ions to the shoots by a rapid transpiration stream in the xylem. We found high salinity to be the main factor inducing growth and found it to be correlated with the high pH effect on proline increase in glycophytes (Elder, Reed) and Weeping Alkaligrass, in contrast to Common Glasswort. We suggest that proline accumulation allows osmotic adjustment in the green parts of reeds and alkaligrasses, but may have another function (in Elder). Common Glasswort accumulates large amounts of Na^+^, which is energetically more effective than proline accumulation for osmotic adjustment. Organic matter affects plant growth and proline levels, but soil salinity and pH alter nutrient availability. Plant distribution along the salinity gradient indicates that Elder is the most salt-sensitive species compared to Reed, Alkaligrass, and Glasswort. Salinity and the lack of control of thick reeds, which compete with other plant groups, affect the distribution of halophytes in saline environments.

## 1. Introduction

Competition among plants in stressful environments favors those species that are able to establish themselves, grow to maturity, and survive to reproduce [1,2,3]. Species with superior tolerance to extremes of salinity, drought, and temperature are the best competitors among other plants at each of the critical stages of the growth cycle [1,4,5,6]. Environmental factors influence the character, composition, growth, and development of individual plants and plant communities [2,7,8]. When these factors exceed the optimum tolerance of a plant, they stress it and, in turn, affect its development and its structural, physiological, and biochemical processes [9,10,11].

The condition of different ecological groups of plants depends significantly on the environmental factors. The degree of this condition is expressed by differences in measurable condition indicators such as stress protein activity (proline Pro), lipoperoxidation intensity (malondialdehyde MDA), and plant shoot length. At the same time, this condition is determined by the content of organic matter and the level of electrolytic conductivity in the rhizosphere zone of these plants [12].

Soils are essential natural resources that provide important ecosystem and ecophysiological functions, such as the medium for plant growth, the regulation and distribution of water flow, and environmental buffers in the formation, attenuation, and degradation of natural and toxic compounds [13,14]. A plant’s tolerance of the environment is chemically dependent and remains in direct dependence on the quality of its buffer elements [7]. At the same time, salts, especially sodium salts, are one of the main physiological threats to the environment. Salinity prevents, limits, or disrupts the normal metabolism, water quality, and nutrient uptake of plants and soil biota [11,15,16]. Soil salinity affects plant growth and development through osmotic stress, especially the deleterious effects of toxic Na^+^ and Cl^−^ and, to some extent, Cl^−^ and SO_4_^2−^, and Mg^2+^, as well as the nutrient imbalance caused by excess Na^+^ and Cl^−^ ions [17,18]. The mechanisms that minimize damage from high salinity vary among plants, and several mechanisms must operate in a coordinated fashion to manage Na^+^ [19,20] and maintain ionic and osmotic homeostasis [5,21,22].

However, during the onset of and change in environmental symptoms, as well as during the further progression of the salt stress development process in plants, all major physiological processes of plants, such as photosynthesis and the protein, carbohydrate, and lipid metabolic pathways, as well as energy metabolism, are directly conditioned by salinity, acidification, and potential environmental redox [5,23,24]. Malondialdehyde (MDA), the end product of thiobarbituric acid degradation and lipid peroxidation, is now recognized as an indicator of pro-oxidative damage to plant cell membranes, manifested by disruption of their overall metabolic functions and the destabilization or complete loss of cellular integrity [25,26,27]. However, lipoperoxidation is significantly correlated with a variety of damage at different levels in plants (cells, tails) caused by numerous and physiologically distinct environmental stressors [23,27,28,29], i.e., salinity [30,31,32], heavy metals [33,34], and drought stress [6]. Thus, cell membrane stability has been widely used to distinguish between salt-tolerant and salt-sensitive variants [35].

At the same time, plants must maintain their internal water potential below that of the soil and maintain turgor and water uptake for growth under salinity stress conditions. This requires an increase in osmotic pressure, either by the uptake of soil solutes or by the synthesis of compatible metabolic solutes [18,29] that do not interfere with normal biochemical reactions [36]. At the same time, proline is abundant in salt-sensitive higher plants and accumulates to a greater extent than other amino acids [37,38,39]. In addition to osmotic regulation, other roles for proline in plant tissues under osmotic stress have been proposed: scavenger of hydroxyl radicals [14,40,41], protection of plasma membrane integrity [42], energy source or energy reduction [43], and source of carbon and nitrogen [22,44].

Many researchers [39,41,45,46,47] investigated a positive correlation between proline accumulation and salt tolerance, in contrast to Aziz et al. [48] and Lutts et al. [49], who stated that the increase in proline under saline conditions was due to salinity injury and was a response to salinity stress rather than an indication of salt tolerance. Glycophytes and halophytes grow differently according to their inability to grow in high salinity environments [10]. In contrast to glycophytes, salt-tolerant halophytes are highly developed and specialized, with well-adapted morphological and physiological traits that allow them to proliferate in high-salinity soils [7,11,16,49]. Of course, we must always bear in mind that the condition of ecological groups of plants depends significantly on environmental factors, and the degree of this condition is expressed in differences in measurable condition indicators (mainly stress protein activity, lipoperoxidation intensity, plant shoot length). At the same time, this condition is determined by the content of organic matter and the level of electrolytic conductivity in the rhizosphere zone of these plants [12].

Many studies have investigated the physiological responses of plants under controlled conditions [18,20,22,29]. However, there is still a need to investigate the relationships between glycophytes and halophytes and the many biotic and abiotic factors in their natural environments. Therefore, the aim of this work was to study the morphological and biochemical varieties of ecological groups of plants: glycophytes (Elder *Sambucus nigra* L. and Common Reed *Phragmites australis* (Cav.) Trin. ex Steud.) and halophytes (Weeping Alkaligrass *Puccinellia distans* (Jacq.) Parl and Common Glasswort *Salicornia europaea* L.) in saline environments in a disturbed anthropogenic region of central Poland. It has been shown that phytosociological communities (patches) dominated by *S. europaea* are found only in regions affected by soda production, which are quite numerous in the specific region we studied [50].

It has also been shown that some species of halophytes have colonized these saline areas as a result of the frequent breakdowns of sodium pipelines and so-called wastewater pipelines and collectors, the source of which is the active soda industry. After the reduction in the environmental activity of the soda works and after reclamation works, halophytes remain in these areas for 2–3 years, but then they vegetate and undergo morphological and physiological changes as a result of the continuous and natural process of washing out various salts from the soil and as a result of the further development (expansion) of glycophytes in these areas [51].

However, the Common Reed is always the dominant species, especially in meadows, salt marshes, and fields, and especially after agrotechnical treatments, but also in numerous and phytosociologically diverse ecotone zones. This species gradually but systematically eliminates light-loving halophytes from their existing habitats and causes their progressive degradation, both morphologically and phytosociologically. Therefore, it is very important to accurately determine the influence of edaphic factors (soil salinity Ec, soil acidity pH, availability of minerals (mainly organic matter)) on the growth and changes of biochemical parameters (indicators) of plants (especially halophytes), i.e., the content of malondialdehyde (MDA) and proline (Pro), in the roots and green parts of both glycophytes and halophytes in their natural habitats (i.e., in the saline areas of the Kujawy region (Central Poland)). Thanks to this, we can assess the extent of their reactions to various environmental stressors and the destabilization of the management of chemical elements, which allows us to analyze their adaptation strategies in the changed environments which shape their condition.

## 2. Study Area; Material and Methods

**Figure 1:**
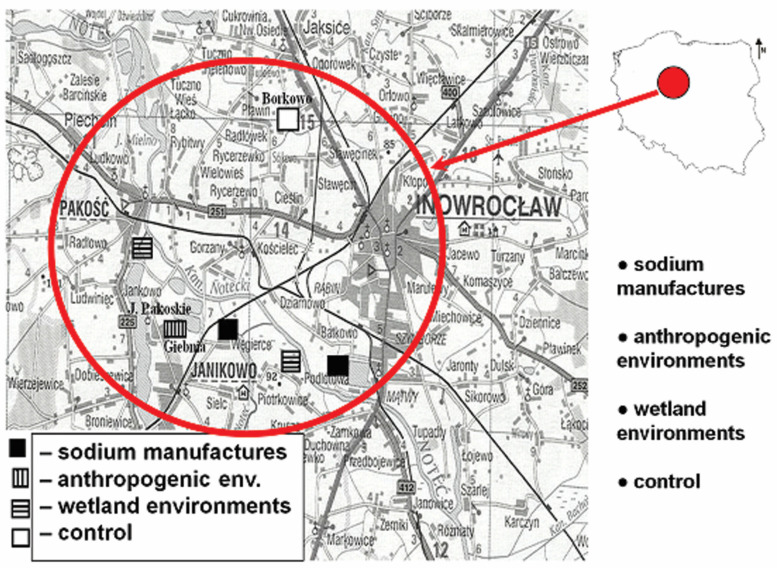
Study area (sodium factories, anthropogenic environments, wetland environments, control) in the Inowrocław Region of Ecological Danger IRED (www.maps.google.pl, modified).

Our studies are innovative in the most naturally saline areas of Europe and were conducted under natural conditions in different environments. The Kujawy region is located in central Poland (52–53° N, 18–20° E) and is one of the driest areas in central Europe [51]. Its climate is transitional between the oceanic climate of western Europe and the continental climate of eastern Europe. This area is closely related to the Permian period, which was marked by the uplift of brine domes and associated brine springs and saline groundwater, which influenced the development of the sodium industry in the Janikowo and Inowrocław areas of the Kujawy region of central Poland (Figure 1). The secondary salinization, and hence alkalization, of these areas is caused by the human economy, which is related to the infiltration of sewage into the soil and the irrigation of grasslands as a result of improper sealing by settlers [52,53,54,55,56,57].

We conducted our research in selected habitats with different anthropogenic disturbances: (1) “sodium production” (Inowrocław and Janikowo, near sodium production plants); (2) “anthropogenic environments” (landfills near Giebnia agroecosystems, adjacent polluted areas; calcium deposits, post-production tanks); (3) “wetland environments” (floodplains near the Noteć River channel and Pakoskie Lakeland and flushing meadows), and (4) “control” (natural, unpolluted environments near Borkowo; 40 km away from the remaining areas); Figure 1.

The field studies were carried out in four consecutive summer seasons (June–July–August) over four years. Plant species were estimated based on the work of Szafer et al. [58] and classified into four ecological groups: glycophytes (land shrub: Elder *Sambucus nigra* (Adoxaceae)); macrophytes: Common Reed *Phragmites australis* (Poaceae); halophytes (faculty grassy halophyte: Weeping Alkaligrass *Puccinellia distans* (Poaceae)); and obligate halophyte: Common Glasswort *Salicornia europaea* (Amaranthaceae). Macrophytes (Common Reed) and the nitrophilous shrub (Elder) are cosmopolitan species with a wide range of ecological tolerance. Common Reed is generally known as an invasive species [58,59,60], which accumulates trace elements more distinctly than other aquatic plants. It thus has been widely used in constructed wetlands for treatment of industrial wastewaters containing heavy metals [61,62,63,64]. Common Glasswort is an obligatory halophyte, which appears only in saline habitats [65], and is known as a succulent [66]. It is one of most saline-tolerant plants in general and is capable of growing under highly saline conditions in the lowest parts of salt marshes [67,68]. Weeping Alkaligrass is a faculty halophyte, which prefers saline areas but is also found in non-saline habitats [65,69,70].

The green plant parts (leaves) and root samples of Elder (n = 69), Common Reed (n = 78), Weeping Alkaligrass (n = 25), and Common Glasswort (n = 22) were collected from the studied environments (the glycophytes), but only some species were collected from the anthropogenic saline areas (the halophytes) due to their natural distribution. Each sample was taken from four highly concentrated areas of a given environment plus the central area of that environment.

The length of the shoots of Common Reed, Weeping Alkaligrass, and Common Glasswort and the length of the annual flowering shoots of Elder individuals were measured to an accuracy of 0.1 cm. Samples were taken from randomly selected plants at midday and stored in cool freezer bags for transport to the laboratory. Shoot samples were then washed three times with deionized water; roots were gently detached from the soil, then washed extensively with tap water to remove soil particles, rinsed three times with deionized water, and immediately stored at –80 °C for further biochemical analysis [35].

Soil samples were taken from the surface layer of the soil (0–5 cm) and from its deeper layer (5–40 cm) according to the methods of Górny and Grüm [71] and Demirezen and Aksoy [72], and on the basis of other studies, according to which heavy metals falling to the soil mainly accumulate in these layers [73,74,75]. These samples were collected in triplicate from each plant species in each plant habitat studied. The samples were then air dried to constant mass at 65 °C, then homogenized and sieved through 1 mm mesh. Each biochemical analysis of a test sample was performed in triplicate.

The free proline content was determined according to Bates et al. [76] using L-proline as a standard. Approximately 0.5 g of frozen plant material was homogenized in 10 mL of 3% aqueous sulpho-salicylic acid. The homogenate was then centrifuged at 13,000× *g* for 10 min. An amount of 2 mL of the supernatant was transferred to a test tube to which 2 mL of glacial acetic acid and 2 mL of freshly prepared acidic ninhydrin solution (1.25 g ninhydrin dissolved in 30 mL glacial acetic acid and 20 mL 6 M orthophosphoric acid) was added. The tubes were incubated in a water bath at 100 °C for 1 h and the reaction was terminated in an ice bath. The tubes were then filled with 4 mL toluene and vortexed for 20 s in a fume cupboard. The tubes were allowed to stand for at least 10 min. The toluene-containing chromophore was aspirated from the aqueous phase and the absorbance of the toluene phase was measured at 520 nm using toluene as a blank. The concentration of proline was expressed as μmol·g^−1^ wet weight.

The intensity of lipid peroxidation was estimated according to the method of Okhawa et al. [77]. Approximately 0.5 g of frozen plant tissue was cut into small pieces, homogenized with 2.5 mL of 5% trichloroacetic acid, and then centrifuged at 10,000× *g* for 15 min at room temperature. Equal volumes of the supernatant and 0.5% thiobarbituric acid in 20% trichloroacetic acid were added to a new tube and incubated at 96 °C for 25 min, then rapidly cooled in an ice bath. After centrifugation at 8000× *g* for 5 min, the absorbance of the supernatant was recorded at 532 nm and 600 nm. The value for non-specific absorption at 600 nm was subtracted. The concentration of MDA was calculated using an extinction coefficient of 155 mM·cm^−1^ and expressed as μmol·g^−1^ wet weight.

The content of organic matter (OM) was determined by the organic carbon method; the percentage of organic carbon was determined by the roasting method described by Szczepańska [78] and Więckowska [79]. Samples of approximately 2 g dry mass were subjected to heat treatment and high temperature treatment (540 °C) in a muffle furnace. The firing time was 6 h. The samples were then collected in a desiccator and weighed with an accuracy of 0.01 g. The reference method was thermogravimetric analysis carried out with a thermal analyzer, i.e., TGA-DTA Simultaneous Thermal Analyzer from Thermal Analysis TA Instruments with SDT 2960. The types of analysis parameters were as follows: the test atmosphere: air with a temperature range of 20–1000 °C and a heating rate of 10 °C·min^−1^. The content of organic matter was determined by deflagration in a hot muffle furnace according to Szczepańska [78] and Więckowska [79].

The electrolytic conductivity (Ec) of soils was determined by the conductivity method in accordance with ISO 11265 + AC1 [80]. The standard number, standard title, and publisher, in the case of ISO 11265 + AC1 are included in the ref. [81]. Amounts of 15 g of homogenized soil and 30 mL of distilled water were mixed in a volumetric flask. The measurement of salinity of organic soils was performed in homogenized soil solutions, in which the ratio of soil dry weight to H_2_O was 1:5. The flask content was shaken for 1 h. Electrolytic conductivity was measured with an electrode in the top layer solution using a conductometer (Elmetron CC-401 type). The measured values of electrolytic conductivity are given as milli-siemens (mS·cm^−1^) or micro-siemens (μS·cm^−1^).

Soil pH was determined in distilled water at a soil solution ratio of 1:2.5 using a potentiometric EPP-3 glass electrode and an Elmetron C-501 pH-meter. The pH of soils was determined by potentiometry according to ISO 10390 [82]. The standard number, standard title, and publisher, in the case of ISO 10390 are included in the ref. [83]. A sample of the soil material (10 g) was placed in a volumetric flask and 25 mL of distilled water was added (active acidity; pH (H_2_O)). The potential acidity, pH (KCl), was determined in the same way, i.e., 10 g of soil and 25 mL of 1 M KCl were placed in a volumetric flask, left for 24 h, and the pH was measured with a pH-meter model CP-501 (Elmetron Co., Zabrze, Poland) using an EPP-3 type electrode (Elmetron).

## 3. Statistical Analysis

Data were analyzed for normal distribution; those that were not normal were analyzed using non-parametric tests (Mann–Whitney U test). Arithmetic means and descriptive statistics were calculated for MDA and proline content in the roots and green parts of plants, with shoot length and soil parameters in the surface and deeper layers (pH, Ec, OM). Since the data did not show a normal distribution, non-parametric tests were used. The significance of differences in MDA and Pro content and differences in shoot length between environments and between species (significance level at *p* < 0.05) and differences between soil parameters in particular environments were examined using ANOVA Kruskal–Wallis tests, followed by multiple Kruskal–Wallis comparisons and Mann–Whitney U tests. The dependence of the content of the investigated parameters in the roots and shoots of plants from natural conditions (pH, Ec, OM) was calculated according to the rank of the Spearman test (*p* < 0.05). Differences between the contents of biochemical parameters in the roots and green parts of plants were calculated by Wilcoxon matched-pairs tests [82,84,85].

## 4. Results

The distributions of plant species and their ecological groups were closely related to the environmental gradients. At the same time, soil parameters (pH, Ec, organic matter) were different for each type of environment studied (Table 1). We also found the highest level of salinity (Ec) in soil samples collected from the root zones of Common Reed, Weeping Alkaligrass, and Common Glasswort in both surface and deeper layers, while the Ec was higher in the anthropogenic environments (surface layer) and in the sodium production areas (deeper layer) from the root zones of Elder. The pH in both soil layers indicated that soils from all types of environments were alkaline, but the effect was stronger (highest pH) in the polluted anthropogenic areas and sodium factories than in the control and wetlands, except for soils collected from the Elder, where the pH (deeper layer) was lowest in the sodium factories (Table 1).

Environments suitable for halophytes in the sodium production areas were richer in organic matter than those in the anthropogenic sites. The content of organic matter in the soils of the glycophytes (Elder, Common Reed) was higher in the control than in the other sites, except for those from the deeper layer, which were higher in the samples of Common Reed from the sodium factories than in the control (Table 1). The differences between the lowest and highest values of Ec of soils from the vicinity of the Common Reed (36.42 mS·cm^−1^ (surface), 35.98 mS·cm^−1^ (deeper)), compared to that of the Elder (1.18 mS·cm^−1^, 0.57 mS·cm^−1^, respectively), may suggest that the Common Reed has a greater ability to adapt to difficult environmental conditions than the Elder. The same comparison between Weeping Alkaligrass (12.97 mS·cm^−1^ (surface), 13.1 mS·cm^−1^ (deeper)) and Common Glasswort (10.56 mS·cm^−1^ (surface), 9.9 mS·cm^−1^ (deeper)) indicates that Weeping Alkaligrass, as a facultative halophyte, can grow under disturbed and saline conditions (lower salinity), but not very high compared to Common Glasswort (as an obligatory halophyte suitable for high salinity). We also found that Common Reed can exist in saline conditions similar to those of Common Glasswort (Table 1).

We found a statistically significant higher level of MDA only in the green parts of Common Glasswort in the anthropogenic environments than in the sodium production areas (Table 2). At the same time, we found negative correlations of MDA with organic matter (surface), pH (deeper), organic matter (deeper), and Ec (deeper) in the green parts of Common Glasswort (Table 2). We found that MDA increased in the roots and green parts of Common Reed in more saline environments (sodium chloride production areas, anthropogenic environments), but the differences between the levels of lipid peroxidation were not significant. We also found no differences in the MDA content of elderberry tissues between the environments studied (Table 2).

We found a higher content of proline in the roots and green parts of glycophytes in polluted anthropogenic environments and in sodium factories than in the wetlands and control (Table 2). The results of our research indicate that Pro in plant tissues increases with increasing soil Ec and pH (Table 2). However, we also found a negative correlation between pH (deeper layer) and proline content in the roots of Elder. The content of proline in the roots of Weeping Alkaligrass was higher in the saline than in the anthropogenic environments, which was opposite to the green parts of this species. Proline content was also correlated with organic matter (surface layer), which was positive (roots of Weeping Alkaligrass) and negative (roots of Common Reed).

We did not investigate differences in the proline content of Common Glasswort between anthropogenic environments and sodium production areas. However, we found positive correlations between Pro and organic matter (deeper layer) in the roots and negative correlations with pH (surface and deeper layer) in the green parts of Common Glasswort (Table 2).

**Figure 2:**
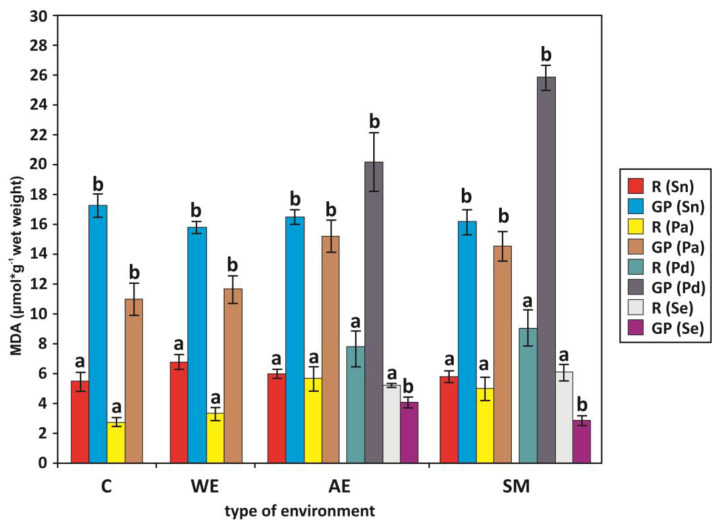
MDA content (μmol·g^−1^ wet weight) in roots (R) and green parts (GP) of Elder *Sambucus nigra* (Sn), Common Reed *Phragmites australis* (Pa), Weeping Alkaligrass *Puccinellia distans* (Pd), and Common Glasswort *Salicornia europaea* (Se) in different environments. Arithmetic means ± SD (standard deviation). Bars with different letters within the same species are different at *p* < 0.05 according to Wilcoxon matched-pairs tests. Environments: C—control, WE—wetland environments, AE—anthropogenic environments, SM—sodium manufactures.

We found a higher level of MDA in the green parts of the plants than in the roots of Elder, Common Reed, and Weeping Alkaligrass in all environments. On the other hand, the degree of lipoperoxidation intensity was higher in the roots than in the green parts of Common Glasswort in the anthropogenic environments and in the sodium production areas (Figure 2). The proline level was higher in the green parts than in the roots of Elder from the anthropogenic environments.

**Figure 3:**
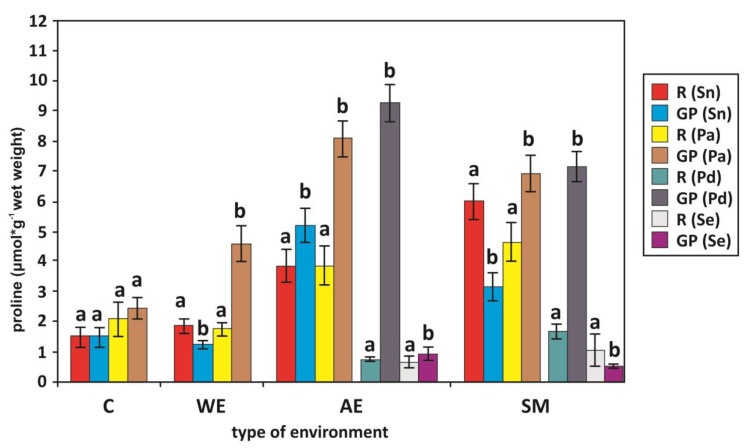
Proline concentration (μmol·g^−1^ wet weight) in the roots (R) and green parts (GP) of Elder *Sambucus nigra* (Sn), Common Reed *Phragmites australis* (Pa), Weeping Alkaligrass *Puccinellia distans* (Pd), and Common Glasswort *Salicornia europaea* (Se) in different environments. Arithmetic means ± SD (standard deviation). Bars with different letters within the same species are significantly different at *p* < 0.05 according to Wilcoxon matched-pairs tests. Environments: C—control, WE—wetland environments, AE—anthropogenic environments, SM—sodium manufactures.

At the same time, we found similar regularities of proline content in Common Reed from wetlands, anthropogenic environments, and sodium factories, and in Weeping Alkaligrass from anthropogenic environments and sodium factories (Figure 3). Proline in Common Glasswort (green parts) was also higher in anthropogenic environments, in contrast to Elder from wetlands and sodium factories (Figure 3, Table 3). We found no differences between proline in the roots and green parts of glycophytes (Common Reed, Elder) from the control and Common Glasswort from the sodium factories (Figure 3, Table 3).

It can be noted that the studied plants differed in their proline content and in the degree of lipoperoxidation in their roots and shoots (Table 3, Figure 2 and Figure 3). We found a significantly higher level of MDA in the roots and green parts of the Elder than in the Common Reed collected from the control and wetlands. The degree of lipid peroxidation was significantly higher in the roots of Weeping Alkaligrass from the sodium production areas than in Common Reed (Table 2 and Table 3).

Our research also showed that MDA was significantly lower in the green parts of Common Glasswort than in the other plants (Elder, Common Reed, Weeping Alkaligrass) in polluted areas (sodium factories, anthropogenic environments). On the other hand, lipid peroxidation was higher in the green parts of Weeping Alkaligrass than in Elder and Common Reed from sodium factories. We also found significantly higher levels of Pro in the green parts of Common Reed than in Elder from the control, wetlands, and sodium factories (Table 2 and Table 3). The content of this organic solution was higher in the roots of glycophytes (Common Reed, Elder) than in halophytes (Weeping Alkaligrass, Common Glasswort). However, the Pro content was lower in the green parts of Common Glasswort than in the other plants studied in anthropogenic environments and in Common Reed and Weeping Alkaligrass from sodium production areas. The Pro content in the green parts of the Weeping Alkaligrass was higher than in the Elder in the sodium factories and in the anthropogenic environments (Table 2 and Table 3).

**Figure 4:**
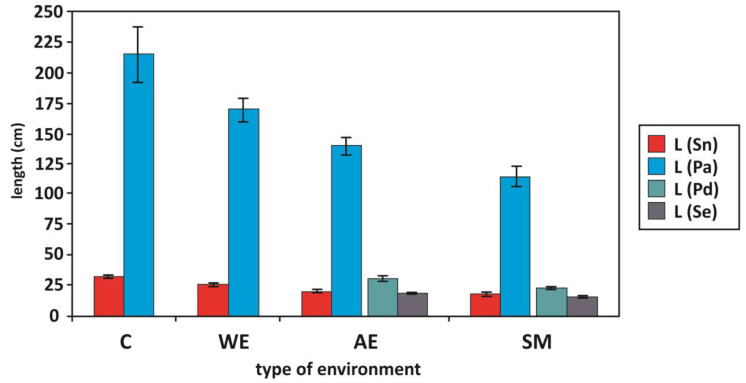
The length of shoots (L; cm) of Elder *Sambucus nigra* (Sn), Common Reed *Phragmites australis* (Pa), Weeping Alkaligrass *Puccinellia distans* (Pd), and Common Glasswort *Salicornia europaea* (Se) in different environments. Arithmetic means ± SD (standard deviation). Bars with different letters within the same species are significantly different at *p* < 0.05 according to Wilcoxon matched-pairs tests. Environments: C—control, WE—wetland environments, AE—anthropogenic environments, SM—sodium manufactures.

We found that the length of the shoots was related to the salinity of the environment. The higher level of Ec in polluted anthropogenic environments and sodium factories inhibited the growth of glycophytes (Common Reed, Elder). We found longer shoots of Elder and Common Reed in the control and wetlands than in the sodium factories and anthropogenic environments (Table 2, Figure 4). Elder shoot length was positively correlated with soil organic matter (deeper and surface layers). Salinity also influenced the growth of Weeping Alkaligrass (r = −0.51, *p* = 0.05); Table 2. At the same time, the shoot length of this species was greater in the less saline anthropogenic environments than in the saline ones. We did not investigate the differences in the shoot length of Common Glasswort between the studied environments. We also found that the MDA of Common Reed and Weeping Alkaligrass and the proline of Elder were negatively correlated with the length of their shoots (Table 2).

## 5. Discussion

Plants living in degraded environments, in particular, are usually exposed to the intense action of multiple and complex stressors in their natural habitats, and environmental stressors cause various unfavorable physiological and biochemical changes in them [5,86], as well as interspecific relationships and changes in their distribution [3,56]. Environmental salinity, soil acidification, and the activity of toxic metals in the catchment area have a destructive aggressive effect on the ecological balance of the soil environment, especially in the rhizosphere, and, at the same time, the activity of numerous aggressive free radicals released by them is very high [8,29,65,73,87].

Reactive oxygen species, especially peroxides and hydroxyl radicals, derived from the biogeochemical transfer of mainly chlorides, nitrates, sulphates, phosphates, and carbonates, aggressively damage the lipids of plant biological membranes as a result of enhanced pro-antioxidant reactions, thus generating lipid peroxidation [23,24,25,26,27,28,29,30,31,32,33,34,35,36,37,38,39,40,41,42,43,44,45,46,47,48,49,50,51,52,53,54,55,56,57,58,59,60,61,62,63,64,65,66,67,68,69,70,71,72,73,74,75,76,77,78,79,80,82,84,85,86,87,88]. However, salt stress of a pro-antioxidant nature may be caused not only by salinity and associated biogeochemical conditions of the substrate, but also by overlapping combinations of additional local soil factors that stimulate plant cell chemistry strategies under these conditions [1,2,28,29].

We did not find differences in the MDA content in the tissues of the glycophytes and Weeping Alkaligrass between the areas studied (this paper), but the average level of lipoperoxidation in the organs of Common Reed and Weeping Alkaligrass was higher in the saline areas than in the control and wetlands (Table 2). These results could be related to the additional activity of unknown, unexplained factors (fertilizers, heavy metals, plant protection products) that disturb the soil balance in less saline habitats (control, wetlands).

It should also be considered that plants are able to mitigate the oxidative damage induced by ROS and have developed a complex antioxidant defense system that includes low-molecular-weight antioxidants as well as antioxidant enzymes, such as superoxide dismutase SOD, catalase CAT, ascorbate peroxidase APx, and glutathione reductase GR, and could thus reduce membrane damage [14,26,89,90]. The report of Eyidogan and Öz [90] confirms that changes in MDA in Chickpea *Cicer arietinum* tissues are associated with significant CAT activity. On the other hand, Lin and Kao [91] indicated that the NaCl treatment of rice leaves did not induce any changes in lipid peroxidation and conductance compared to the control. This was probably due to the lack of accumulation of H_2_O_2_ in the leaves. They also found higher activities of SOD, APx, peroxidase Pox, and GR in the NaCl-stressed rice leaves than in the controls. However, Sudhakar et al. [92] found no changes in the tolerant cultivar in leaves of Mulberry *Morus alba* under NaCl salinity, whereas MDA increased significantly in the sensitive cultivar. They suggested the relative tolerance of the tolerant cultivar based on the low rate of lipid peroxidation and the high constitutive activity of SOD, CAT, POx, GR, and glutathione S-transferase GST.

We found a significantly higher level of lipoperoxidation only in the green parts of the Common Glasswort in the less saline anthropogenic environments (Ec = 32.73 mS (surface layer), Ec = 30.82 mS (deeper layer)) than in the more saline sodium factories (43.29 mS (surface layer), Ec = 40.72 mS (deeper layer)). At the same time, we found that MDA was negatively correlated with the soil parameters pH (deeper layer), organic matter (surface and deeper layer), and Ec (deeper layer); Table 2. We can therefore assume that Common Glasswort, as an obligate halophyte, is adapted to the high degree of salinity of the environment, in contrast to less saline areas with the influence of toxic metals (Pb, Cd) in waste dumps and agricultural cultivation. On the other hand, research by Aghasleh et al. [93] showed that MDA in the shoots of *Salicornia europaea* and *S. persica* remained close to the control at moderate NaCl concentrations (100 and 200 mM). However, they noted an increase in lipoperoxidation in the shoots of these halophytes at higher salinities, which was associated with the loss of chlorophylls under salinity stress. This leads to photoinhibition or ROS formation [26,94].

Based on the salinity conditions for Common Reed and Common Glasswort (Table 1), we can assume that the reed could adapt to a high biodiversity of terrestrial environments. However, we found that the degree of lipoperoxidation was higher in the green parts of glycophytes (Common Reed and Elder) and also in the halophyte Weeping Alkaligrass than in the obligate halophyte (Common Glasswort) in both polluted habitats we studied (Table 2 and Table 3). This suggests that the green parts of glycophytes and Weeping Alkaligrass are more sensitive to environmental stress than those of Common Glasswort. This also confirms the strong adaptation of Common Glasswort to exist in highly saline and alkaline soils.

It is also interesting to note that the MDA of Common Reed (roots and green parts) is lower than that of Weeping Alkaligrass, despite the higher salinity in the rhizosphere of Common Reed (Table 1, Table 2 and Table 3). Furthermore, many reports [58,59,60,61,62,63,64,65,66,67,68,69,70,71,72,73,74,75,76,77,78,79,80,82,84,85,86,87,88,89,90,91,92,93,94,95] confirm the ability of Common Reed to rapidly dominate marsh plant communities (e.g., in the USA). It is one of the most abundant plant species in US coastal wetlands and is considered an indicator of wetland disturbance [95].

The presence of Common Reed affects the environment, including effects such as altered edaphic conditions, increased vertical accretion of marsh substrates, changes in floral diversity, altered nutrient cycling, and changes in animal populations depending on the taxa [96]. Some authors do not currently consider Common Reed to be an invasive plant [97]. Reed canary-grass also has a broad salinity tolerance (180 mM) and maintains a lower Na^+^:K^+^ ratio by an effective mechanism that excludes Na^+^ in its tissues [98]. Furthermore, we found a higher degree of lipid peroxidation in the green parts than in the roots of Elder, Common Reed, and Weeping Alkaligrass, in contrast to the same relationship in Common Glasswort in all the environments studied in this paper (Figure 2). This may indicate that the green parts may be more sensitive to environmental disturbances than the roots. Furthermore, Na^+^ and other ions play an important role in this process and are transported to the shoots by a rapidly moving transpiration stream in the xylem and can accumulate to higher levels in the shoots than in the roots [15,18,73,74,98]. Roots tend to maintain relatively constant NaCl levels and can regulate NaCl by exporting it to the soil or to the shoots. There is limited evidence for the extensive recirculation of Na^+^ from shoots to roots, suggesting that Na^+^ transport is largely unidirectional, leading to the progressive accumulation of Na^+^ as leaves age [19,98].

Our research indicates that the glycophytes Elderberry and Common Reed can accumulate proline in their roots and green parts in more saline habitats, i.e., in sodium and anthropogenic environments (Table 2). The lack of differences in proline accumulation between the tissues of these glycophytes from the control sites confirms that changes in proline in plant organs could be related to the effect of stressors. Soil alkalization is also related to the proline content in tissues of Common Reed (roots, green parts) and Elder (green parts), because of the positive relationships between pH and proline (Table 2). At the same time, Shi and Sheng [99] indicated that synergism between salinity and alkalinity caused even stronger responses (higher proline levels in shoots of Sunflower *Helianthus annuus*) than salinity and pH as single factors.

Similarly, osmotic and ion toxic effects depend on salt concentration, whereas pH effects depend on buffer capacity, which in turn is closely related to both alkalinity and salinity stress. Thus, higher alkalinity and greater buffer capacity make environmental adaptation more difficult for plants [7,14,29,99]. However, soil pH was probably not the main factor causing proline accumulation in the roots of Elderberry (this paper).

As it is clear from our studies, higher proline levels are found in the green parts of Common Reed from the control, wetlands, and sodium factories, and of Weeping Alkaligrass from the sodium and anthropogenic sites, than in the green parts of Elder (Table 3), compared with higher Pro content in the green parts than in the roots of Common Reed and Weeping Alkaligrass, in contrast to Elder (Figure 3). This may suggest a different function (scavenger of hydroxyl radicals, protection of plasma membrane integrity, sink of energy or reducing power, source of carbon and nitrogen) of proline in Elderberry tissues. It is likely that proline accumulation in the organs of Common Reed and Weeping Alkaligrass is related to their osmotic adaptation and could be an indicator of salinity tolerance. At the same time, Zhu et al. [100] suggested that osmoregulation by both organic solutes (proline, betaine) and inorganic ions (K^+^) is an important factor in the adaptation of Common Reed to drought and salinity.

In contrast to glycophytes, halophytes have to tolerate the high salinity of their habitats and therefore have to absorb water from a soil solution with low water potential [4,101]. However, research by Kozłowski et al. [102] showed that the diversity of the organic and mineral components of the green parts of Weeping Alkaligrass collected near the Janikowo Sodium Factory (Central Poland) was relative to the high soil conductivity. The individuals were divided by the quantity of crops of the green parts of those living in favorable (5.25 mS·cm^−1^) and unfavorable (23.83 mS·cm^−1^) conditions. The first group formed a crop three times larger than the second and contained more proteins and minerals and also 170% more Na^+^ ions. This may indicate that Weeping Alkaligrass can accumulate not only organic but also inorganic osmo-protectants.

At the same time, Khan et al. [4] showed that the moderately salt-tolerant halophyte Griffith’s Saltbush *Atriplex griffithii v. stocksii* accumulates 40% of its dry weight in ions to achieve a negative water potential gradient, but can also accumulate glycine betaine as an osmo-protectant. The energetic cost of organic solute synthesis for osmoregulation is very high [19]. Therefore, it is advantageous if these processes are maintained mainly by the accumulation of inorganic ions that can be easily taken up from the soil [4,11,23,101]. Meanwhile, we noticed the lack of significant differences in the Pro levels in the tissues of Common Glasswort from the anthropogenic sites and sodium factories and the lower Pro levels in the green parts of all species studied, except of Elder in the sodium factories (Table 2 and Table 3). This may indicate that Common Glasswort does not accumulate proline in response to the increasing salinity of sodium factories.

Many studies indicate that the tendency to accumulate high amounts of NaCl in shoots is particularly associated with dicotyledonous halophytes, and in these plants NaCl may account for almost the entire osmotic potential of the shoots (i.e., NaCl is preferentially used as an osmotic factor) [5,18,19,20,22,102,103]. Balnokin et al. [66] also confirm that the water content in the leaves and roots of *Salicornia* sp. was hardly affected by NaCl, even at extremely high NaCl concentrations in monocultures. On the other hand, Aghaleh et al. [93] found a higher proline content in the shoots of Common Glasswort with increasing salinity. The accumulation of this osmolyte has been demonstrated in various abiotic stress plants [104].

In our research, it is probable that the influence of factors other than salinity (waste dumps) in anthropogenic environments caused an increase in proline content in the green parts of Weeping Alkaligrass compared to those from sodium factories and in the green parts of Common Glasswort compared to its roots (Table 2). We also did not find any differences in proline content in the green parts of Elder and Common Glasswort in more saline environments (sodium factories), in contrast to other species (Table 3). Furthermore, soil organic matter could influence proline levels in plant tissues. This relationship is positive in the roots of Weeping Alkaligrass and Common Glasswort and negative in the green parts of Weeping Alkaligrass and the roots of Common Reed (Table 2). This suggests that salinity and higher pH may affect nutrient uptake and that this process is likely to be species dependent.

The results of our research also confirm that an increase in soil salinity causes growth inhibition (Elder, Common Reed, Weeping Alkaligrass) as an example of the morphological symptoms of the damaging effects of salinity stress (Table 2, Figure 4). We also did not find any differences in the length of the shoots of Common Glasswort between the investigated environments (Table 2). Other reports confirm that low [93] and moderate [66] NaCl concentrations in monocultures stimulate the shoot growth of Common Glasswort. However, the extent of growth depends on the ability of the plant to cope with the osmotic and toxic effects of salt, and the shoot growth of Common Glasswort is reduced at higher soil salinities [66]. At the same time, research by de Lacerda at al. [105] found that the shoot development of two genotypes (salt-tolerant: CSF 20, salt-sensitive: CSF 18) of Sorghum *Sorghum bicolor* was inhibited by salinity. This inhibition was greater in the sensitive than in the tolerant genotypes and the difference between the genotypes increased with time. This result was confirmed by Shi and Sheng [99] who showed that the development of relative tolerance to salt in Sunflower *Helianthus annuus* is inhibited with increasing salinity and, in addition, soil pH, whereas at low salinity and low pH the growth of sensitive plants is only moderately inhibited or even stimulated by salt stress.

In contrast to salinity, organic matter content is only significantly positively correlated with shoot length in Elder (this paper). Thus, salinity was probably the main factor affecting growth, but it could also be the factor of nutrient availability. Furthermore, studies by Merino et al. [106] and Zhao et al. [16] indicated that increasing nutrient availability did not allow plants to better tolerate salinity stress. The response of Saltmeadow Cortgrass *Spartina patens* growth to nutrient availability varies with salinity, such that soil properties (organic matter) have a greater effect on plant growth at lower salinities than at higher salinities. Furthermore, increasing nutrient availability has no significant effect on Saltmeadow Cortgrass except in non-saline areas [106]. At the same time, Tester and Davenport [19], as well as Gulmezoglu and İzci [20], Erdine et al. [18], Yadav et al. [5], and Zhu et al. [22], showed that high Na^+^ inhibits the uptake of other nutrients by interfering with transporters in the roots, such as K^+^-selective ion channels, and by reducing root growth with high Na^+^ concentration.

As shown in our studies (Table 2), the negative correlation of shoot length with MDA content (Common Reed, Weeping Alkaligrass) and proline content (Elder) could be related to the distribution of energy requirements for shoot growth. On the other hand, Jain et al. [107] found that an increase in proline content correlated with the inhibition of fresh weight gain in the Groundnut *Arachis hypogaea*. Furthermore, the extent of lipid peroxidation, as reflected by MDA, is significantly reduced under similar salinity stress conditions [39]. These findings suggest links with the synthesis of organic solutes for osmoregulation or macromolecular protection, and for maintaining membrane integrity and regulating ion concentration.

The above considerations and the results of our research confirm that the presence of plants in the environment and their phytosociological variability are their sensitive responses to progressive changes in the constituent biogeochemical factors of their habitats. This environmental variability determines the growth rates and condition of plants, as well as the changing distribution of different ecological groups of plants in their phytosociological complexes. The differences between the electrolytic conductivity (Ec) of the soil and the roots of the plant species studied by us in the anthropogenic environments, in the region of the sodium production factories, and the mutual correlations with the changes in growth and biochemical indicators in the tissues of these plants, indicate that the most important factor determining the occurrence and condition of plants in such environments is salinity, followed by acidity (pH) and organic matter content. These environmental factors determine the condition and distribution of plants (Table 1, Figure 2, Figure 3 and Figure 4).

As shown by the results of our current research and other studies [11,16,108], the physiological responses of plants to Ec, pH, and organic matter content are strongly species dependent. In addition, it can be assumed that Common Glasswort, unlike other species we have studied (e.g., Weeping Alkaligrass, Elder, Common Reed), only shows an adaptation to development under conditions of extreme salinity. However, clear changes in soil conditions (especially soil permeability and the ability to strongly predict ions), as well as the lack of control over the expansion of expansive plant species (Common Reed) as a result of meadow cutting and grazing [56,69], may influence the distribution of halophytes in anthropogenically degraded environments.

It should also be noted that the changes in plant biochemical parameters (MDA, proline) found by us differed quite significantly from the analytical results described for plants subjected to artificial stress under experimental laboratory conditions [1,2,93]. In conclusion, we can say that the results of our research, carried out under the natural conditions of degraded and saline environments, are important in interpreting the ecophysiological responses of different ecological groups of plants to destabilizing factors in anthropogenic and naturally saline environments. At the same time, however, these factors may have limited local significance for plants, because many factors acting simultaneously on plants and interacting with each other act on plants in the natural environment in different ways, and each time it depends on the ecophysiological situation of a given habitat [15,99,100,109,110]. Further detailed environmental studies are therefore needed to compare analogous results obtained under experimental conditions with those from anthropogenic, industrial, and naturally destabilized (especially naturally saline) sites.

## 6. Conclusions

1. The differences among the soil Ec of the roots of plants in saline environments and the relationship with the extent of growth changes and biochemical parameters in plants suggest that salinity is the most important factor affecting the performance and distribution of ecological plant groups. The type of environment is important to explain the physiological responses of plants to the action of degradation.
2. The intensity of lipoperoxidation, proline content, and growth of halophytes (Weeping Alkaligrass, Common Glasswort) and salt-glycophytes (Common Reed, Elder) are mainly influenced by edaphic pH, Ec, and organic matter. The physiological responses of these plants are highly species dependent.
3. The higher salinity induced the growth of glycophytes and Weeping Alkaligrass, in contrast to Common Glasswort.
4. The increase in proline osmo-protectant in the glycophytes and the facultative halophyte (Weeping Alkaligrass), in contrast to Common Glasswort, is determined by a higher soil Ec and pH.
5. Lipid peroxidation can be determined by environmental disturbance, which influenced the higher MDA in shoots of halophytes (Common Glasswort) and the whole body of other species studied in less saline areas. The green parts of Elder, Common Reed, and Weeping Alkaligrass are more resistant to environmental disturbance than those of Common Glasswort.
6. Organic matter determines halophyte and glycophyte growth and proline content, but high soil salinity and pH are associated with nutrient availability.
7. Salinity and the lack of control of thick reeds, leading to increased competition, affect the distribution of halophytes in saline environments.

## Figures and Tables

**Table 1:** Edaphic conditions: pH, organic matter (OM), and salinity (Ec) in soil surface (s) and soil deeper (d) layer from root zone (rhizosphere) of glycophytes and halophytes from different habitats. Arithmetic means ± SD (standard deviation). Values designated by upper index (type of environment) within a column differ significantly at *p* ≤ 0.05.

Environment	pH (s)			OM (s)			Ec (s)			pH (d)			OM (d)			Ec (d)		
	Mean	±	SD	Mean	±	SD	Mean	±	SD	Mean	±	SD	Mean	±	SD	Mean	±	SD
**Elder *Sambucus nigra***																		
Control C	8.01 ^WE^	±	0.21	14.65 ^WE,AE,SM^	±	3.09	1.31 ^WE,AE,SM^	±	0.10	8.27 ^SM^	±	0.15	9.19 ^WE,AE,SM^	±	1.76	1.2 ^SM^	±	0.12
Wetland W	7.81 ^C,AE,SM^	±	0.07	6.11 ^C,AE^	±	3.22	1.58 ^C,AE,SM^	±	0.32	8.35 ^SM^	±	0.22	5.26 ^C^	±	3.15	1.14 ^AE.SM^	±	0.55
Anthropogenic AE	8.02 ^WE^	±	0.18	11.73 ^C,WE,SM^	±	5.02	2.49 ^C,WE^	±	0.53	8.57 ^SM^	±	0.39	4.14 ^C^	±	0.79	1.35 ^WE^	±	0.21
Sodium manufactures SM	8.37 ^WE^	±	0.45	5.99 ^C,AE^	±	1.57	2.14 ^C,WE^	±	0.32	7.41 ^C,WE,AE^	±	0.74	4.46 ^C^	±	1.88	1.71 ^C.WE^	±	0.35
**Common Reed *Phragmites australis***																		
Control C	7.8 ^WE,SM^	±	0.23	15.23 ^WE,AE,SM^	±	4.70	2.44 ^AE,SM^	±	0.85	8.12 ^WE,AE,SM^	±	0.16	9.27 ^WE,AE^	±	2.29	1.59 ^AE,SM^	±	0.55
Wetland W	8.06 ^C^	±	0.26	4.78 ^C^	±	2.25	1.61 ^AE,SM^	±	0.30	8.46 ^C^	±	0.19	2.55 ^C,AE,SM^	±	2.08	1.29 ^AE,SM^	±	0.52
Anthropogenic AE	8.00	±	0.37	5.88 ^C,SM^	±	2.29	30.1 ^C,WE,SM^	±	3.53	8.47 ^C^	±	0.48	4.43 ^C,WE,SM^	±	1.63	26 ^C,WE,SM^	±	4.55
Sodium manufactures SM	9.05 ^C^	±	1.62	4.05 ^C,AE^	±	1.36	38.03 ^C,WE,AE^	±	6.57	9.2 ^C^	±	1.52	10.06 ^WE,AE^	±	3.07	37.27 ^C,WE,AE^	±	1.05
**Weeping Alkaligrass *Puccinellia distans***																		
Anthropogenic AE	9.55 ^SM^	±	0.48	4.25 ^SM^	±	1.19	4.48 ^SM^	±	0.01	9.70	±	1.14	3.25 ^SM^	±	0.03	5.83 ^SM^	±	0.04
Sodium manufactures SM	9.44 ^AE^	±	0.04	5.10 ^AE^	±	1.38	17.45 ^AE^	±	1.51	10.13	±	0.24	7.91 ^AE^	±	1.58	18.93 ^AE^	±	1.16
**Common Glasswort *Salicornia europaea***																		
Anthropogenic AE	7.6 ^SM^	±	0.05	3.02 ^SM^	±	0.20	32.73 ^SM^	±	1.31	7.69 ^SM^	±	0.05	3.37 ^SM^	±	0.05	30.82 ^SM^	±	0.74
Sodium manufactures SM	8.06 ^AE^	±	1.35	9.60 ^AE^	±	2.35	43.29 ^AE^	±	6.61	8.45 ^AE^	±	1.26	6.85 ^AE^	±	2.45	40.72 ^AE^	±	1.43

**Table 2:** Arithmetic means ± SD (standard deviation) of malondialdehyde (MDA), proline (Pro), and length (L) of shoots of selected plant species from different environments. Values designated by the upper index (name of environment) within a column differs at *p* ≤ 0.05. Dependences in MDA and Pro content in roots and shoots and length of shoots of plants between environments with soil parameters (acidity pH, salinity Ec, organic matter OM) were calculated by correlation coefficient (r) according to the rank of the Spearman test (significance level at *p* < 0.05); s—soil surface layer, d—soil deeper layer.

Environment	Element	Mean	±	SD	Relations	Mean	±	SD	Relations
**glycophytes**									
		**Elder** ***Sambucus nigra***				**Common Reed *Phragmites australis***			
**green parts**									
Control C	MDA	17.24	±	2.59		10.98	±	3.40	Ec (d) 0.28 *
Wetland W		15.83	±	1.93		11.66	±	4.45	L −0.4 ***
Anthropogenic AE		16.50	±	2.13		15.23	±	5.29	
Sodium manufactures SM		16.16	±	3.31		14.54	±	4.32	
Control C	Pro	1.47 ^AE^	±	1.12	pH (s) 0.55 ***	2.42 ^AE,SM^	±	1.19	Ec (s) 0.48 ***
Wetland W		1.22 ^AE.SM^	±	0.68	Ec (s) 0.66 ***	4.60	±	3.00	pH (d) 0.39 ***
Anthropogenic AE		5.22 ^C,WE^	±	2.31	Ec (d) 0.54 ***	8.08 ^C^	±	2.93	Ec (d) 0.48 ***
Sodium manufactures SM		3.13 ^WE^	±	1.84	L −0.25 *	6.93 ^C^	±	2.72	
Control C	L	31.64 ^AE,SM^	±	4.30	OM (s) 0.25 *	214.7 ^AE,SM^	±	70.22	MDA −0.4 ***
Wetland W		25.36 ^SM^	±	6.87	Ec (s) −0.51 ***	169.71 ^SM^	±	47.43	Ec (s) −0.4 ***
Anthropogenic AE		21.12 ^C^	±	4.61	OM (d) 0.41 ***	139.92 ^C^	±	34.32	Ec (d) −0.37 ***
Sodium manufactures SM		18.44 ^WE,C^	±	5.60	Pro −0.25 *	115.24 ^WE.C^	±	41.13	
**roots**									
Control C	MDA	5.44	±	1.89		2.76	±	0.92	Ec (s) 0.23 *
Wetland W		6.74	±	2.52		3.29	±	2.11	Ec (d) 0.27 *
Anthropogenic AE		5.97	±	1.09		5.64	±	4.10	
Sodium manufactures SM		5.75	±	1.58		4.95	±	3.73	
Control C	Pro	1.49 ^AE,SM^	±	1.01	pH (s) 0.55 ***	2.06	±	1.82	OM (s) −0.29 **
Wetland W		1.83 ^AE,SM^	±	1.15	Ec (s) 0.54 ***	1.73 ^SM^	±	1.13	Ec (s) 0.42 ***
Anthropogenic AE		3.83 ^WE,C^	±	2.11	pH (d) −0.37 **	3.80	±	3.66	pH (d) 0.29 **
Sodium manufactures SM		6.03 ^WE,C^	±	2.37	Ec (d) 0.37 **	4.63 ^WE^	±	3.09	Ec (d) 0.42 ***
**halophytes**									
		**Weeping Alkaligrass *Puccinellia distans***				**Common Glasswort *Salicornia europaea***			
**green parts**									
Anthropogenic AE	MDA	20.28	±	6.94	L −0.5 *	4.02 ^SM^	±	1.02	OM (s) −0.53 *
Sodium manufactures SM		25.93	±	2.68		2.78 ^AE^	±	0.81	pH (d) −0.45 *
									OM (d) −0.57 *
									Ec (d) −0.53 **
Anthropogenic AE	Pro	9.28 ^SM^	±	2.32	Ec (s) −0.43 *	0.94	±	0.75	pH (d) −0.58 **
Sodium manufactures SM		7.17 ^AE^	±	1.55		0.48	±	0.13	
Anthropogenic AE	L	29.85 ^SM^	±	8.09	MDA −0.5 *	18.25	±	2.80	
Sodium manufactures SM		22.3 ^AM^	±	3.47	Ec (s) −0.51 *	15.38	±	3.34	
**roots**									
Anthropogenic AE	MDA	7.68	±	4.53		5.14	±	0.85	
Sodium manufactures SM		9.04	±	3.84		6.04	±	1.57	
Anthropogenic AE	Pro	0.72 ^SM^	±	0.29	Ec (s) 0.75 ***	0.62	±	0.70	OM (d) 0.46 *
Sodium manufactures SM		1.66 ^AE^	±	0.81	OM (s) 0.65 ***	1.07	±	1.35	
					Ec (d) 0.65 ***				

Particular relationships (“Relations”) are measured by correlation coefficients (r): * (0.5–0.7), ** (0.7–0.9), *** (0.9–1.0) [84,85].

**Table 3:** Differences among malondialdehyde (MDA) and proline (Pro) content in plants from the same type of environment (*p* < 0.005); Elder *Sambucus nigra* (Sn), Common Reed *Phragmites australis* (Pa), Weeping Alkaligrass *Puccinellia distans* (Pd), Common Glasswort *Salicornia europaea* (Se).

Environment	Green Parts		Roots	
	MDA	Pro	MDA	Pro
Control	Pa:Sn **	Pa:Sn *	Pa:Sn **	
Wetland	Pa:Sn ***	Pa:Sn ***	Pa:Sn ***	
Anthropogenic	Sn, Pa, Pd:Se ***	Sn:Pd, Se*		Sn:Pd, Se* **
		Pa, Pd:Se ***		Pa:Pd **, Se ***
Sodium manufactures	Sn:Pd, Se **	Sn:Pa **, Pd *	Pa:Pd **	Sn:Pd, Se ***
	Pa:Pd ***, Se *	Pa, Pd:Se ***		Pa:Pd *, Se **
	Pd:Se ***			

Differences between the examined indices (MDA, Pro) in plants in different environments are expressed as a measure of the strength of their relationship between the analyzed plant species. These relationships are measured by correlation coefficients (r): * (0.5–0.7), ** (0.7–0.9), *** (0.9–1.0) [84,85].

## Data Availability

Material data are available from the authors of the paper.

## References

[1] Akhtar M.S. (2019). Salt Stress, Microbes, and Plant Interactions: Mechanisms and Molecular Approaches.

[2] Grzesiak M.T., Rzepka A., Hura T., Grzesiak S. (2019). Plant Functioning Under Environmental Stress.

[3] Silva L.C.R., Lambers H. (2020). Soil-plant-atmosphere interactions: Structure, function, and predictive scaling for climate change mitigation. Plant Soil.

[4] Khan M.A., Ungar I.A., Showalter A.M. (2000). Effects of salinity on growth, water relations and ion accumulation of the subtropical perennial halophyte, *Atriplex griffithii* var. stocksii. Ann. Bot..

[5] Yadav B., Jogawat A., Rahman M.S., Narayan O.P. (2021). Secondary metabolites in the drought stress tolerance of crop plants: A review. Gene Rep..

[6] Zia R., Nawaz M.S., Siddique M.J., Hakim S., Imran A. (2021). Plant survival under drought stress: Implications, adaptive responses, and integrated rhizosphere management strategy for stress mitigation. Microbiol. Res..

[7] Amir R., Munir F., Kubra G., Nauman I., Noor N., Akhtar M.S. (2019). Role of Signaling Pathways in Improving Salt Stress in Plants. Salt Stress, Microbes, and Plant Interactions: Mechanisms and Molecular Approaches.

[8] Neema P., Jisha K.C. (2020). Physiological and Biochemical Responses of *Aerva lanata* (L.) Juss. ex Schult. under Heavy Metal Stress. J. Stress Physiol. Biochem..

[9] Jaleel C.A., Gopi R., Manivannan P., Panneerselvam R. (2007). Antioxidative potentials as a protective mechanism in *Catharanthus roseus* (L.) G. Don. plants under salinity stress. Turk. J. Bot..

[10] Manivannan P., Jaleel C.A., Sankar B., Somasundaram R., Murali P.V., Sridharan R., Panneerselvam R. (2007). Salt stress mitigation by calcium chloride in *Vigna radiate* (L.) Wilczek. Acta Biol. Cracov. Ser. Bot..

[11] Azzeme A.M., Abdullah S.N.A., Akhtar M.S. (2019). Adaptive Mechanisms of Plants Against Salt Stress and Salt Shock. Salt Stress, Microbes, and Plant Interactions: Mechanisms and Molecular Approaches.

[12] Schulze E.D., Beck E., Mçller-Hohenstein K. (2005). Plant Ecology.

[13] Franzluebbers A.J. (2002). Soil organic matter stratification ratio as an indicator of soil quality. Soil Tillage Res..

[14] Bhattacharyya M., Sing P.S., Patni B. (2020). Effect of Salinity Stress on Growth Performance, Cellular Responses and Antioxidant Production Capacity of Medicinal Plants. J. Plant Sci. Res..

[15] Jones R.J.A., Montanarella L. (2002). Contributions to the International Workshop “Land Degradation”.

[16] Zhao C., Zhang H., Song C., Zhu J.-K., Shabala S. (2020). Mechanisms of Plant Responses and Adaptation to Soil Salinity. Innovation.

[17] Sairam R.K., Tyagi A. (2004). Physiology and molecular biology of salinity stress tolerance in plants. Curr. Sci..

[18] Erdine C., Inal B., Erez E., Ekincial A., Sensoy S. (2021). Comparative Adaptation Responses of Melon (*Cucumis melo* L.) Genotypes to Salinity Stress. J. Agr. Sci. Tech..

[19] Tester M., Davenport R. (2003). Na^+^ tolerance and Na^+^ transport in higher plants. Ann. Bot..

[20] Gulmezoglu N., İzci E. (2020). Ionic responses of bean (*Phaseolus vulgaris* L.) plants under salinity stress and humic acid applications. Not. Bot. Horti Agrobot..

[21] Borsani O., Valpuesta V., Botella M.A. (2003). Developing salt tolerant plants in a new century: A molecular biology approach. Plant Cell, Tissue Org. Culture.

[22] Zhu H., Zhao J., Gong L. (2021). The morphological and chemical properties of fne roots respond to nitrogen addition in a temperate Schrenk’s spruce (*Picea schrenkiana*) forest. Sci. Rep..

[23] Parida A.K., Das A.B. (2005). Salt tolerance and salinity effect on plants: A review. Ecotoxicol. Environ. Saf..

[24] Skórzyńska-Polit E. (2007). Lipid peroxidation in plant cells, its physiological role and changes under heavy metal stress. Acta Soc. Bot. Pol..

[25] Scandalios J.G. (1993). Oxygen stress and superoxide dismutase. Plant Physiol..

[26] Kong W., Liu F., Zhang C., Zhang J., Feng H. (2016). Non-destructive determination of Malondialdehyde (MDA) distribution in oilseed rape leaves by laboratory scale NIR hyperspectral imaging. Sci. Rep..

[27] Sairam R.K., Srivastava G.C., Saxena D.C. (2000). Increased antioxidant activity under elevated temperature: A mechanism of heat tolerance in wheat genotypes. Biol. Plant..

[28] Elkahoui S., Hernandez J.A., Abdelly C., Ghrir R., Limam F. (2005). Effects of salt on lipid peroxidation and antioxidant enzyme activities of *Catharanthus roseus* suspension cells. Plant Sci..

[29] Song X., Wang Y., Lv X. (2016). Responses of plant biomass, photosynthesis and lipid peroxidation to warming and precipitation change in two dominant species (*Stipa grandis* and *Leymus chinensis*) from North China Grasslands. Ecol. Evol..

[30] Alché J. (2019). de Dios. A concise appraisal of lipid oxidation and lipoxidation in higher plants. Redox Biol..

[31] Gossett D.R., Millhollon E.P., Lucas M.C. (1994). Antioxidant response to NaCl stress in salt-tolerant and salt sensitive cultivars of cotton. Crop Sci..

[32] Dionisio-Sese M.L., Tobita S. (1998). Antioxidant responses of rice seedlings to salinity stress. Plant Sci..

[33] Sreenivasulu N., Ramanjulu S., Ramachandra-Kini K., Prakash H.S., Shekar-Shetty H., Savitri H.S., Sudhakar C. (1999). Total peroxidase activity and peroxidase isoforms as modified by salt stress in two cultivars of fox-tail millet with differential salt tolerance. Plant Sci..

[34] Loureiro S., Santos C., Pinto G., Costa A., Monteiro M., Nogueira A.J.A., Soares A.M.V.M. (2006). Toxicity assessment of two soils from Jales Mine (Portugal) using plants: Growth and biochemical parameters. Arch. Environ. Contam. Toxicol..

[35] Bidar G., Pruvot C., Garçon G., Verdin A., Shirali P., Douay F. (2009). Seasonal and annual variations of metal uptake, bioaccumulation, and toxicity in *Trifolium repens* and *Lolium perenne* growing in a heavy metal-contaminated field. Environ. Sci. Pollut. Res..

[36] Meloni D.A., Oliva M.A., Martinez C.A., Cambraia J. (2003). Photosynthesis and activity of superoxide dismutase, peroxidase and glutathione reductase in cotton under salt stress. Environ. Exp. Bot..

[37] Zhifang G., Loescher W.H. (2003). Expression of a celery mannose 6-phosphate reductase in *Arabidopsis thaliana* enhances salt tolerance and induces biosynthesis of both mannitol and a glucosyl-mannitol dimmer. Plant Cell Environ..

[38] McCue K.F., Hanson A.D. (1990). Drought and salt tolerance: Towards understanding and application. Trends Biotech..

[39] Abraham E., Rigo G., Szekely G., Nagy R., Koncz C., Szabados L. (2003). Light-dependent induction of proline biosynthesis by abscisic acid and salt stress is inhibited by brassinosteroid in *Arabidopsis*. Plant Mol. Biol..

[40] Vasilakoglou I., Dhima K., Giannakoula A., Dordas C., Skiada V., Papadopoulou K. (2021). Carbon Assimilation, Isotope Discrimination, Proline and Lipid Peroxidation Contribution to Barley (*Hordeum vulgare*) Salinity Tolerance. Plants.

[41] Smirnoff N., Cumbes Q.J. (1989). Hydroxyl radical scavenging activity of compatible solutes. Phytochem.

[42] Hong Z., Lakkineni K., Zhang Z., Verma D.P.S. (2000). Removal of feedback inhibition of D1-pyrroline–5-carboxylate synthetase results in increased proline accumulation and protection of plants from osmotic stress. Plant Physiol..

[43] Mansour M.M.F. (1998). Protection of plasma membrane of onion epidermal cells by glycinebetaine and proline against NaCl stress. Plant Physiol. Biochem..

[44] Verbruggen N., Hua X.-J., May M., van Montagu M. (1996). Environmental and developmental signals modulate proline homeostasis: Evidence for a negative transcriptional regulator. Proc. Nat. Acad. Sci. USA.

[45] Ahmad I., Hellebust J.A. (1988). The relationship between inorganic nitrogen metabolism and proline accumulation in osmoregulatory responses of two euryhaline microalgae. Plant Physiol..

[46] Fougere F., Le Rudulier D., Streeter J.G. (1991). Effects of salt stress on amino acid, organic acid, and carbohydrate composition of roots, bacteroids, and cytosol of alfalfa (*Medicago sativa* L.). Plant Physiol..

[47] Nanjo T., Fujita M., Seki M., Kato T., Tabata S., Shinozaki K. (2003). Toxicity of free proline revealed in an Arabidopsis T-DNA-tagged mutant deficient in proline dehydrogenase. Plant Cell Physiol..

[48] Aziz A., Martin-Tanguy J., Larher F. (1998). Stress induced changes in polyamine and tyramine levels can regulate proline accumulation in tomato leaf discs treated with sodium chloride. Physiol. Plant..

[49] Lutts S., Majerus V., Kinet J.M. (1999). NaCl effects on proline metabolism in rice (*Oryza sativa*) seedlings. Physiol. Plant..

[50] Khan M.A., Duke N.C. (2001). Halophytes—A resource for the future. Wetl. Ecol. Mgmt..

[51] Piernik A. (2006). Growth of the three meadow species along a salinity gradient in an inland saline habitat: Transplant experiment. Pol J. Ecol..

[52] Piernik A. (2003). Inland halophilous vegetation as indicator of soil salinity. Basic Appl. Ecol..

[53] Cieśla W., Dąbkowska-Naskręt H., Siuda W. (1981). Stan zasolenia gleb w okolicach Inowrocławskich Zakładów Sodowych w Mątwach. Roczn. Glebozn..

[54] Cieśla W., Dąbkowska-Naskręt H. (1984). Właściwości zasolonych gleb w sąsiedztwie Janikowskich Zakładów Sodowych na Kujawach. Roczn. Glebozn..

[55] Czerwiński Z., Pracz J., Piątek A. (1984). Wpływ odpadów z Janikowskich Zakładów Sodowych na tereny rolnicze. Roczn. Glebozn..

[56] Czerwiński Z. (1996). Zasolenie wód i gleb na terenie Kujaw. Roczn. Glebozn..

[57] Ferreira C.S.S., Seifollahi-Aghmiuni S., Destouni G., Ghajarnia N., Kalantari Z. (2022). Soil degradation in the European Mediterranean region: Processes, status and consequences. Sci. Total Environ..

[58] Szafer W., Kulczyński S., Pawłowski B. (1986). Rośliny Polskie.

[59] Windham L., Lathrop R.G. (1999). Effects of *Phragmites australis* (Common Reed) Invasion on Aboveground Biomass and Soil Properties in Brackish Tidal Marsh of the Mullica River, New Jersey. Estuaries.

[60] Ederli L., Reale L., Ferranti F., Pasqualini S. (2004). Responses induced by high concentration of cadmium in *Phragmites australis* roots. Physiol. Plant..

[61] Próchnicki P. (2005). The expansion of common reed (*Phragmites australis* (Cav.) Trin. ex. Steud.) in the anastomosing river valley after cessation of agriculture use (Narew River Valley, NE Poland). Pol. J. Ecol..

[62] Dunbabin J.S., Bowmer K.H. (1992). Potential use of constructed wetlands for treatment of industrial wastewaters containing metals. Sci. Total Environ..

[63] Aksoy A., Duman F., Sezen G. (2005). Heavy metal accumulation and distribution in narrow-leaved cattail (*Typha angustifolia*) and common reed (*Phragmites australis*). J. Freshw. Ecol..

[64] Duman F., Cicek M., Sezen G. (2007). Seasonal changes of metal accumulation and distribution in common club rush (*Schoenoplectus lacustris*) and common reed (*Phragmites australis*). Ecotoxicology.

[65] Ghassemzadeh F., Yousefzadeh H., Arbab-Zavar M.H. (2008). Removing arsenic and antimony by *Phragmites australis*: Rhizofiltration technology. J. Appl. Sci..

[66] Wilkoń-Michalska J. (1963). The halophytes from Kujawy. Stud. Soc. Sci. Torun. D Bot..

[67] Balnokin Y.V., Myasoedov N.A., Shamsutdinov Z.S., Shamsutdinov N.Z. (2005). Significance of Na^+^ and K^+^ for sustained hydration of organ tissues in ecologically distinct halophytes of the family *Chenopodiaceae*. Russ. J. Plant Physiol..

[68] Ungar I.A. (1977). The relationship between soil water potential and plant water potential in two inland halophytes under field conditions. Bot. Gaz..

[69] Egan T.P., Ungar I.A. (2000). Mortality of the Salt Marsh Species *Salicornia europaea* and *Atriplex prostrata* (*Chenopodiaceae*) in Response to Inundation. Ohio J. Sci..

[70] Wilkoń-Michalska J. (1970). Plant succession in the halophyte reserve in Ciechocinek between 1954 and 1965. Ochr. Przyr..

[71] Górny M., Grüm L. (1993). Methods in Soil Zoology.

[72] Demirezen D., Aksoy A. (2006). Common hydrophytes as bioindicators of iron and manganese pollutions. Ecol. Indic..

[73] Prasad M.N.V., Sajwan K.S., Naidu R. (2006). Trace Elements in the Environment. Biogeochemistry, Biotechnology, and Bioremediation.

[74] Kabata-Pendias A., Pendias H. (2010). Trace Elements in Soils and Plants.

[75] Kabata-Pendias A., Szteke B. (2019). Trace Elements in Abiotic and Biotic Environments.

[76] Bates L.S., Waldren R.P., Teare I.D. (1973). Rapid determination of free proline for water stress studies. Plant Soil.

[77] Ohkawa H., Ohishi N., Yagi Y. (1979). Assay of lipid peroxides in animal tissue by thiobarbituric acid reaction. Analyt. Biochem..

[78] Szczepańska W. (1996). Metody Instrumentalne w Analizie Chemicznej.

[79] Więckowska J. (1997). Termiczna Analiza Różnicowa i Termograwimetria.

[80] Otterson D.W. (2015). Tech Talk: (10) Electrolytic Conductivity Measurement Basics. Meas. Control.

[81] Slovenski Standard SIST ISO 11265:1996; Soil Quality—Determination of the Specific Electrical Conductivity. ICS: 13.080.20 Fizikalne Lastnosti tal Physical Properties of Soils. 2003-01. iTeh Standard Preview (Standards.iteh.ai). Slovenski Inštitut za Standardizacijo. Razmnoževanje Celote ali delov tega Standarda ni Dovoljeno. Int. Org. for Standardization, Case Postale 56. CH-l 211 Geneve 20, Switzerland. https://standards.iteh.ai/catalog/standards/sist/4131c4d8-9227-4219-b9c6-1fe89c83a45d/sist-iso-11265-1996.

[82] Bieganowski A., Cieśla J. (2014). Electrochemical Measurements in Soils. Encyclopedia of Agrophysics.

[83] International Standard ISO 10390. 2021; Soil, Treated Biowaste and Sludge—Determination of pH. ISO Copyright Office CP 401, Ch. de Blandonnet 8, CH-1214 Vernier, Geneva, Switzerland. iTeh Standard Preview (Standards.iteh.ai) ISO 10390:2021. https://standards.iteh.ai/catalog/standards/sist/f2ca1637-a0cb-47a8-a868-cd68a9defd91/iso-10390-2021.

[84] Zar J.H. (1998). Biostatistical Analysis.

[85] Hill T., Lewicki P. (2006). Statistics: Methods and Applications: A Comprehensive Reference for Science, Industry and Data Mining.

[86] Stanisz A. (2006). The Accessible Course of the Statistics with the use Statistica.pl on Examples from the Medicine.

[87] Çakırlar H., Çiçek N., Fedina I., Georgieva K., Doğru A., Velitchkova M. (2008). NaCl induced cross-acclimation to UV-B radiation in four Barley (*Hordeum vulgare* L.) cultivars. Acta Physiol. Plant..

[88] Kabata-Pendias A., Mukherjee A.B. (2007). Trace Elements from Soil to Human.

[89] Imlay J.A. (2003). Pathways of oxidative damage. Ann. Rev. Microbiol..

[90] Foyer C.H., Noctor G. (2000). Oxygen processing in photosynthesis: Regulation and signalling. New Phytol..

[91] Eyidogan F., Öz M.T. (2007). Effect of salinity on antioxidant responses of chickpea seedlings. Acta Physiol. Plant..

[92] Lin C.C., Kao C.H. (2000). Effect of NaCl stress on H2O2 metabolism in rice leaves. Plant Growth Reg..

[93] Sudhakar C., Lakshmi A., Giridarakumar S. (2001). Changes in the antioxidant enzyme efficacy in two high yielding genotypes of mulberry (*Morus alba* L.) under NaCl salinity. Plant Sci..

[94] Aghaleh M., Niknam V., Ebrahimzadeh H., Razavi K. (2009). Salt stress effects on growth, pigments, proteins and lipid peroxidation in *Salicornia persica* and *S. europaea*. Biol. Plant..

[95] Kato M., Shimizu S. (1985). Chlorophyll metabolism in higher plants, V.I. Involvement of peroxidase in chlorophyll degeneration. Plant Cell Physiol..

[96] Rudrappa T., Bonsall J., Gallagher J.L., Seliskar D.M., Bais H.P. (2007). Root-secreted allelochemical in the noxious weed *Phragmites australis* deploys a reactive oxygen species response and microtubule assembly disruption to execute rhizotoxicity. J. Chem. Ecol..

[97] Meyerson L.A., Saltonstall K., Windham L., Kiviat E., Findlay S. (2000). A comparison of *Phragmites australis* in freshwater and brackish marsh environments in North America. Wet. Ecol. Mgmt..

[98] Acevedo-Rodríguez P., Strong M.T. (2012). Catalogue of seed plants of the West Indies. Smithson. Contrib. Bot..

[99] Woźny A. (2004). Komórka w Warunkach Stresu Środowiskowego.

[100] Vives-Peris V., López-Climent M.F., Pérez-Clemente R.M., Gómez-Cadenas A. (2020). Root Involvement in Plant Responses to Adverse Environmental Conditions. Agronomy.

[101] Shi D., Sheng Y. (2005). Effect of various salt-alkaline mixed stress conditions on sunflower seedlings and analysis of their stress factors. Environ. Exp. Bot..

[102] Zhu X., Jing Y., Chen G., Wang S., Zhang C. (2003). Solute levels and osmoregulatory enzyme activities in reed plants adapted to drought and saline habitats. Plant Growth Reg..

[103] Flowers T.J., Troke P.F., Yeo A.R. (1977). The mechanism of salt tolerance in halophytes. Ann. Rev. Plant Physiol..

[104] Kozłowski S., Golinski P., Zielewicz W., Lembicz M., Rogowski A. (2004). Changes in the chemical composition of spreading meadow-grass (*Puccinellia distans* L. Parl.) against the influence of salinity as anthropogenical factor. Ann. UMCS.

[105] Glenn E.P., Brown J.J., Blumwald E. (1999). Salt tolerance and crop potential of halophytes. Crit. Rev. Plant Sci..

[106] Ozden M., Demirel U., Kahraman A. (2009). Effects of proline on antioxidant system in leaves of grapevine (*Vitis vinifera* L.) exposed to oxidative stress by H_2_O_2_. Sci. Hortic..

[107] Lacerda C.F., Cambraia J., Oliva M.A., Ruiz H.A. (2003). 2003 Osmotic adjustment in roots leaves of two sorghum genotypes under NaCl stress Brazilian. J. Plant Physiol..

[108] Merino J.H., Dayna Huval Ć., Andy Ć., Nyman J. (2010). Implication of nutrient and salinity interaction on the productivity of *Spartina patens*. Wet. Ecol. Mgmt..

[109] Jain M., Mathur G., Koul S., Sarin N.B. (2001). Ameliorative effects of proline on salt stress-induced lipid peroxidation in cell lines of groundnut (*Arachis hypogaea* L.). Plant Cell Rep..

[110] Silvestri S., Defina A., Marani M. (2005). Tidal regime, salinity and salt marsh plant zonation. Estauarine Coast. Shelf Sci..

